# CLARITY and PACT-based imaging of adult zebrafish and mouse for whole-animal analysis of infections

**DOI:** 10.1242/dmm.021394

**Published:** 2015-12-01

**Authors:** Mark R. Cronan, Allison F. Rosenberg, Stefan H. Oehlers, Joseph W. Saelens, Dana M. Sisk, Kristen L. Jurcic Smith, Sunhee Lee, David M. Tobin

**Affiliations:** 1 Department of Molecular Genetics and Microbiology, Duke University School of Medicine, Durham, NC 27710, USA; 2 Duke Human Vaccine Institute andDepartment of Medicine, Duke University School of Medicine, Durham, NC 27710, USA; 3 Department of Immunology, Duke University School of Medicine, Durham, NC 27710, USA

**Keywords:** Zebrafish, Infection, PACT, CLARITY, Imaging, Mouse, Tuberculosis, Mycobacteria

## Abstract

Visualization of infection and the associated host response has been challenging in adult vertebrates. Owing to their transparency, zebrafish larvae have been used to directly observe infection *in vivo*; however, such larvae have not yet developed a functional adaptive immune system. Cells involved in adaptive immunity mature later and have therefore been difficult to access optically in intact animals. Thus, the study of many aspects of vertebrate infection requires dissection of adult organs or *ex vivo* isolation of immune cells. Recently, CLARITY and PACT (passive clarity technique) methodologies have enabled clearing and direct visualization of dissected organs. Here, we show that these techniques can be applied to image host-pathogen interactions directly in whole animals. CLARITY and PACT-based clearing of whole adult zebrafish and *Mycobacterium tuberculosis*-infected mouse lungs enables imaging of mycobacterial granulomas deep within tissue to a depth of more than 1 mm. Using established transgenic lines, we were able to image normal and pathogenic structures and their surrounding host context at high resolution. We identified the three-dimensional organization of granuloma-associated angiogenesis, an important feature of mycobacterial infection, and characterized the induction of the cytokine tumor necrosis factor (TNF) within the granuloma using an established fluorescent reporter line. We observed heterogeneity in TNF induction within granuloma macrophages, consistent with an evolving view of the tuberculous granuloma as a non-uniform, heterogeneous structure. Broad application of this technique will enable new understanding of host-pathogen interactions *in situ*.

## Abstract (precis)

**Summary:** The authors apply the CLARITY and PACT techniques to image infectious structures located deep within whole adult zebrafish and mouse organs, enabling visualization of mycobacterial granulomas and host response without sectioning.

## INTRODUCTION

Owing to the limited optical clarity of vertebrate tissue, analysis of host immune cell interactions with pathogenic organisms has generally focused on either *ex vivo* analysis by flow cytometry or traditional pathology techniques on thin sections. These techniques provide only limited spatial information, resulting in the loss of much of the three-dimensional context of infection.

New model systems have emerged to enable the direct imaging of host-pathogen interactions. The optically clear zebrafish larva has provided a useful vertebrate model of bacterial, fungal and viral infections (Brothers et al., 2011; Palha et al., 2013; Gabor et al., 2014; Goody et al., 2014; Gratacap and Wheeler, 2014; Torraca et al., 2014). Conservation of the framework of the zebrafish immune system with that of the mammalian immune system has allowed ready translation of findings in zebrafish to mice and humans (Tobin et al., 2010, 2012; Adams et al., 2011; Hall et al., 2013; van der Vaart et al., 2014). Although early infection events are easily visualized in larval zebrafish, three distinct pigment-producing cells – xanthophores, iridophores and melanophores – limit the ability to image infection in adults (Rawls et al., 2001). Even animals treated with phenylthiourea, which inhibits melanization, do not retain transparency into adulthood. As in mammals, zebrafish adaptive immunity develops later (in zebrafish by 4 weeks post-fertilization), making examination of these responses in larvae impossible (Lam et al., 2004; Hess and Boehm, 2012; Renshaw and Trede, 2012).

A zebrafish strain called *casper*, which lacks melanophores and iridiphores, has been constructed through the combination of two mutations: *nacre* (a mutant allele of the *mitfa* gene) and *roy orbison*, whose molecular identity is unknown (White et al., 2008). This strain allows live imaging of infection in adults (Whipps et al., 2014); however, imaging in *casper* is still complicated by light scattering within intact tissue. The genetics of the *casper* mutant also present challenges to the use of established transgenes and mutants, because these elements must first be crossed into the background of the two *casper* mutations. The genetic background of *casper* might also complicate the study of immunity. Neither of the mutants involved in *casper* have been fully characterized with respect to immune function, but pigmentation mutants in zebrafish, mice and humans have been associated with a number of immunodeficiencies (Stinchcombe et al., 2004; Kaplan et al., 2008; Levesque et al., 2013). The mammalian homolog of the *mitfa* gene mutated in the *casper* line has also been associated with changes in immune signaling (Yannay-Cohen et al., 2009; Smith et al., 2014a; Gutknecht et al., 2015). Thus, it is important to identify methods that will allow characterization of microbial pathogenesis in a standard genetic background that is easily compatible with immune marker lines.

The difficulty in imaging through dense tissues has been attributed in part to scattering of light owing to the high index of refraction of cellular lipids within the tissue (Chung et al., 2013; Richardson and Lichtman, 2015). Recently, the CLARITY technique and PACT (passive clarity technique) have been described, allowing visual access to intact organs (Chung et al., 2013; Tomer et al., 2014; Yang et al., 2014). Tissue morphology is maintained by crosslinking cellular proteins and DNA in acrylamide-containing hydrogel solutions, and lipid-mediated light scattering is minimized by removing cellular lipids (Chung et al., 2013; Yang et al., 2014). This approach has been used to permit optical access to diverse tissues, including brain, heart, kidney and lung (Chung et al., 2013; Yang et al., 2014).

In the zebrafish, a number of host reporter lines have been generated that include macrophages, neutrophils, B cells and T cells (Langenau et al., 2004; Renshaw et al., 2006; Hall et al., 2007; Ellett et al., 2011; Page et al., 2013), as well as fluorescent reporters of cytokine production (Palha et al., 2013; Marjoram et al., 2015). Here, we combine fluorescent transgenic reporter lines with the CLARITY and PACT techniques to visualize cell and cytokine localization within whole adult zebrafish infected with *Mycobacterium marinum* (*M. marinum*). We find that CLARITY and PACT clear iridophores and xanthophores and reduce melanocyte pigmentation intensity, enabling microscopy within intact adult zebrafish. We are able to image these cleared zebrafish to depths in excess of 1 mm and examine host-pathogen interactions using fluorescent bacteria and zebrafish transgenic lines. We extended this approach to mouse lungs, where we found that PACT-mediated clearing allows us to visualize fluorescent *Mycobacterium tuberculosis* (*M. tuberculosis*) in three dimensions within intact lung tissue.

**RESOURCE IMPACT**

**Background**

Imaging of biological tissue can provide key insight into diverse physiological processes with relevance to disease. However, imaging of many optically inaccessible tissues within an intact organism has proven challenging. Technological advances, including more sophisticated microscopes, brighter genetically encoded fluorophores and the use of transparent organisms, such as zebrafish larvae, have improved the imaging of complex physiological processes. However, the study of host-microbe interactions in the context of infection frequently involves techniques that provide limited spatial information and models that are unsuitable for visualizing the adaptive immune system. The recently described hydrogel-embedding techniques CLARITY and PACT allow visual access to diverse, deep tissues while preserving tissue architecture and fluorescence of genetically encoded fluorophores. To date, the techniques have generally been applied for imaging of dissected organs.

**Results**

In this study, the authors modify the CLARITY and PACT techniques to enable clearing of whole adult zebrafish. Adult zebrafish possess three distinct pigment lineages that limit imaging within these animals, restricting their use in studies of the vertebrate adaptive immune system. The authors show that CLARITY and PACT reduce or eliminate these pigments, allowing imaging to depths in excess of 1 mm in whole animals. Mycobacterial infection results in the formation of a characteristic host structure called a granuloma. The authors use CLARITY and PACT to image bacterial localization within zebrafish granulomas. Using characterized fluorescent reporter zebrafish lines, the authors visualize two processes that are crucial to mycobacterial pathogenesis – cytokine induction and vascularization of the granuloma – in intact animals. Their analysis reveals considerable heterogeneity within the granuloma. Finally, the authors apply these techniques to *Mycobacterium tuberculosis* infection in mouse lungs, enabling three-dimensional visualization of infection using a BSL-3 pathogen.

**Implications and future directions**

This study shows that CLARITY and PACT can be used to clear adult zebrafish tissue and mouse organs for imaging of microbial pathogenesis in a native context. Studies of infection in zebrafish have largely focused on larvae, owing to their optical clarity. The approach described herein, based on adaptation of CLARITY and PACT, enables imaging of adult zebrafish infection models *in toto*, providing a powerful tool for the exploration of host-pathogen interactions in the context of a mature immune system. These techniques are compatible with the array of reporter lines in mouse and zebrafish, enabling the investigation of diverse infectious disease processes within complex local host environments.

## RESULTS

### A method for adapting CLARITY and PACT to intact adult zebrafish

We sought to apply clearing techniques to intact adult zebrafish infected with *M. marinum* in order to directly visualize mycobacterial infection within the context of a functional adaptive immune system. The CLARITY method achieves tissue clearing through either a rapid, active clearing process driven by electrophoresis within detergent solutions or a slower, passive clearing process in which tissue is cleared by incubation in detergent alone (Chung et al., 2013; Tomer et al., 2014). As the active clearing approach requires specialized equipment and can cause tissue damage through excessive heating, we sought to devise a method to passively clear intact adult zebrafish (Chung et al., 2013; Tomer et al., 2014). Chung et al. (2013) described the use of a hydrogel solution containing 4% acrylamide/0.05% bis-acrylamide, which is used to passively clear a zebrafish brain in 15 days. We initially attempted to use a 4% acrylamide/0.05% bis-acrylamide hydrogel solution as well as a 4% acrylamide solution that omitted bis-acrylamide to hydrogel-embed and passively clear whole adult zebrafish. However, after about a month of clearing, whole adult zebrafish embedded in these hydrogel solutions were incompletely cleared and we observed tissue damage at later time points. Tomer et al. (2014) found that decreased acrylamide concentrations could speed passive clearing of tissue and suggested the use of a range of acrylamide/bis-acrylamide concentrations from 4% acrylamide/0.05% bis-acrylamide to 0.5% acrylamide/0.0125% bis-acrylamide, depending on the application and rate of clearing desired. We next tested 1% acrylamide/0.05% bis-acrylamide and found that we could passively clear whole adult zebrafish in about a week.

**Fig. 1.**
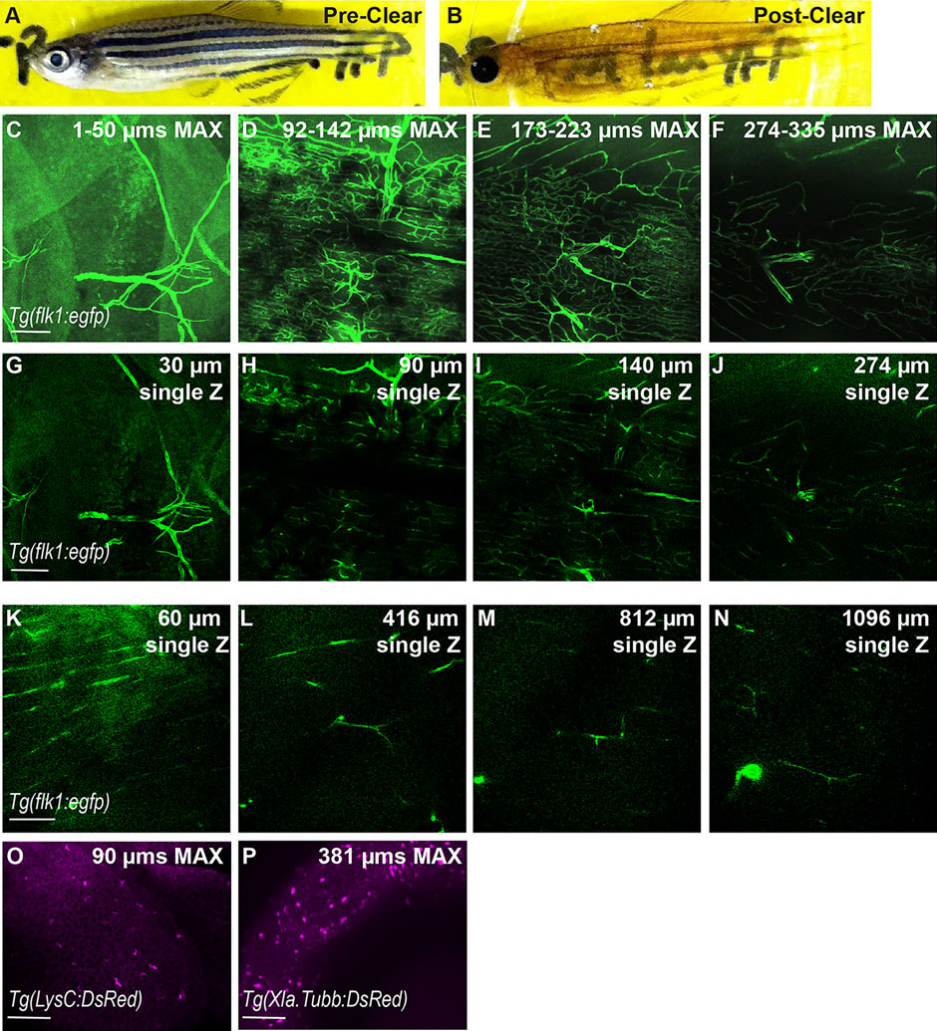
**CLARITY protocol adapted for imaging intact zebrafish adults.** (A,B) Zebrafish adult pre- and post-clearing. (C-N) Whole-body CLARITY allows imaging of fluorescent vasculature deep within the adult body. Blood vessels labeled by *Tg(flk1:egfp)* are imaged from the surface to deep within while maintaining fluorescence intensity and resolution. (C-J) Individual images obtained using an SP8 confocal microscope, ranging from the animal's scales (surface=1 µm) to 335 µm deep. (C-F) Z-stack is split into ∼50 µm maximum projection images to allow for clear views of vascular structures. (G-J) Individual Z planes from stack. (K-N) Individual images from two-photon microscopy ranging from the animal's scales (surface=1 µm) to >1 mm deep. 1-µm optical sections are shown. Scale bars: 100 µm. Single Z frames were exported and gamma adjusted in FIJI/ImageJ for increased visibility, with all gamma adjustments applied uniformly across all images. (O,P) CLARITY techniques are compatible with red fluorescent proteins. (O) Neutrophils within the epidermis were imaged using the transgenic line *Tg(LysC:DsRed)*. 90-μm maximum projection image. (P) Neuronal cell bodies within the eye of cleared zebrafish in a 381-μm maximum projection from the transgenic line *Tg(Xla.Tubb:DsRed)*.

**Fig. 2.**
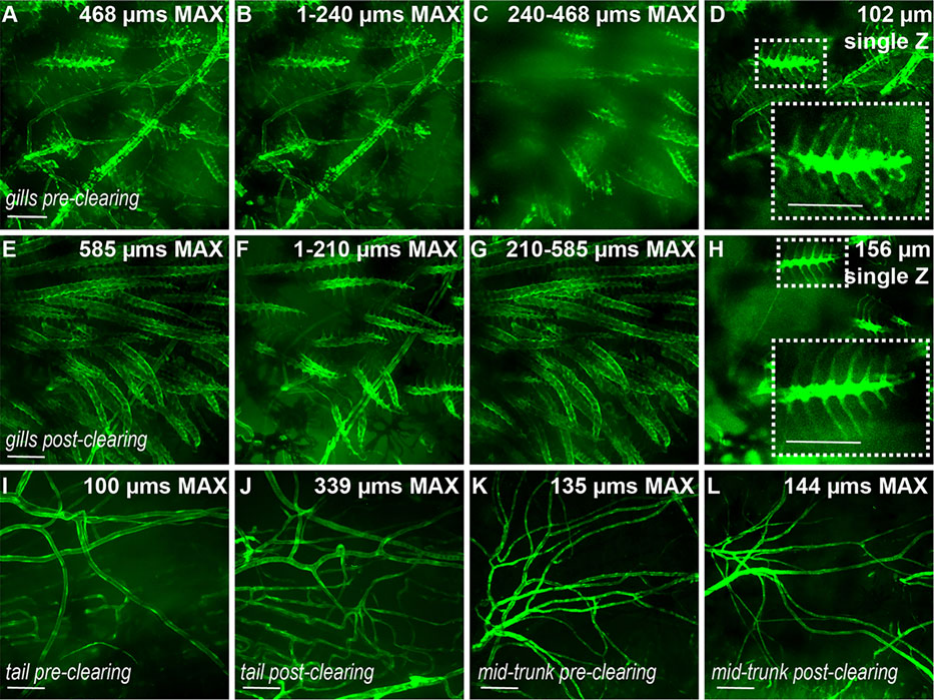
**PACT protocol maintains integrity of blood vessels.** (A-D) Blood vessels in gills labeled by *Tg(flk1:egfp)* in a zebrafish adult pre-clearing (immediately post-euthanasia) and the same animal (E-H) post-clearing. Increasing numbers of gill blood vessels are visible deeper within the body following clearing (G compared to C). Single Z frames (D,H, and insets) demonstrate that fine structures are unaffected by the clearing process. (I,J) Blood vessels in the tail of the same animal pre- and post-clearing. (K,L) Large blood vessels in the mid-trunk of the same animal pre- and post-clearing. Scale bars: 120 µm. Single Z frames were exported and gamma adjusted in FIJI/ImageJ for increased visibility, with all gamma adjustments applied uniformly across all images.

The clearing protocol removed iridophores and xanthophores completely, and greatly diminished the intensity of melanocytes (Fig. 1A,B). To assess whether we could still detect fluorescence within established transgenic lines, we cleared the vascular transgenic line *Tg(kdrl:egfp)*^s843^ [hereafter referred to as *Tg(flk1:egfp)*] (Jin et al., 2005). Using *Tg(flk1:egfp)*, we detected complex vascular networks in adult zebrafish (Fig. 1C-N). We were able to image at least as deep as 1 mm below the skin by conventional 1-photon spinning disk microscope (Fig. 1C-J, and Movie 1) or with 2-photon microscopy (Fig. 1K-N, and Movie 2), indicating that deep imaging was possible with a range of microscopy setups. GFP fluorescence perdured for at least 1 month. To assess background, we also cleared and imaged non-transgenic wild-type zebrafish. As has been reported in a number of organisms owing to the presence of elastin, we detected modest autofluorescence within some blood vessel walls (Deyl et al., 1980), but minimal autofluorescence outside of the vasculature (Fig. S1). The GFP fluorescence in *Tg(flk1:egfp)* animals was markedly higher than any detected vessel autofluorescence, enabling dramatically deeper imaging with greater resolution (Figs 1 and 2).

To confirm that this technique was compatible with diverse fluorophores, we also cleared animals from two separate DsRed-based transgenic lines, *Tg(Xla.Tubb:DsRed)^zf148^* (Peri and Nüsslein-Volhard, 2008), which labels neurons, and *Tg(LysC:DsRed)^nz50^* (Hall et al., 2007), which labels neutrophils. Using these lines, we were able to visualize neutrophils in the epidermis and neural cell bodies in the eye, respectively, indicating that structurally distinct fluorophores remain intact through the clearing process (Fig. 1O-P).

### Whole-animal clearing retains tissue and blood-vessel integrity

We next assessed whether the clearing process itself led to alterations in organ or vascular morphology. Direct imaging of superficial adult zebrafish vasculature has been previously demonstrated (Huang et al., 2003; Xu et al., 2014). Thus, we imaged blood vessels from the same animal before and after clearing of freshly euthanized adult *Tg(flk1:egfp)* animals to assess whether the process compromised native morphology or organization. To establish blood-vessel morphology before clearing, we first imaged the vasculature at multiple superficial locations, including the gills, skin of the mid-trunk and fins (Fig. 2), and then processed the animal for clearing. We found that the architecture of blood vessels was preserved following clearing. Additionally, tissue clearing enabled deeper, higher-resolution visualization at these sites, including enhanced visualization of gill blood vessels and vessels in the tail and mid-trunk (Fig. 2). In all cases, we found that vessel architecture was maintained, indicating that tissue clearing did not disrupt vessel morphology.

We next dissected the *Tg(flk1:egfp)* animal imaged in Fig. 2, removing the intestines and the brain (Fig. S2A,D). These organs retained their morphology, and the isolation of dissected organs simplified imaging of specific structures, enabling more rapid and focused assessment of specific areas of interest. Indeed, dissected organs could be visualized quickly by epifluorescence rather than confocal microscopy in relatively thin organs of interest, including vasculature within the intestine (Fig. S2B,C). For thicker organs such as the brain, epifluorescence microscopy enabled limited analysis of vascularization, but the greater thickness and lipid content of the brain made confocal imaging more appropriate. Confocal imaging of vasculature throughout the brain demonstrated tight networks of blood vessels (Fig. S2E-J). These data indicate that diverse organs within the animal can be cleared without distortion using the whole-animal clearing approach. Furthermore, we find that, after clearing and whole-animal imaging, *post hoc* dissection of organs also provides added utility.

### Whole-animal visualization of angiogenesis in mycobacterial infection

**Fig. 3.**
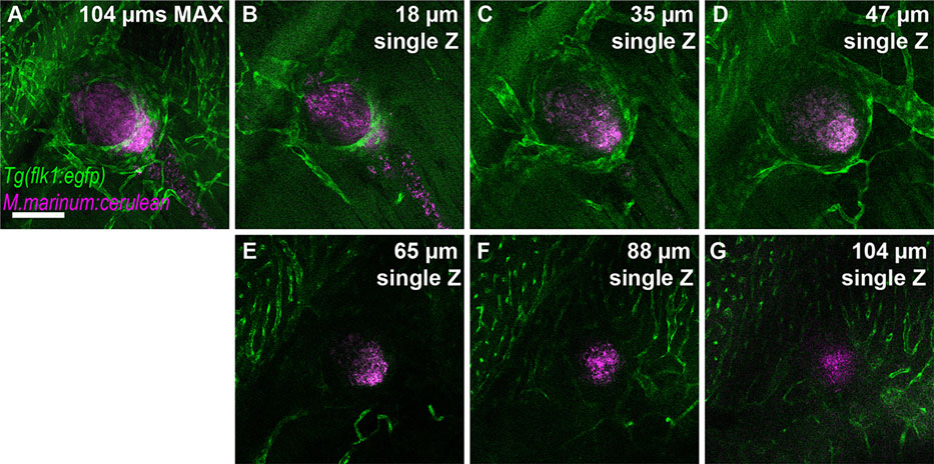
**Granuloma-induced angiogenesis and mycobacterial granulomas within intact adult zebrafish.** (A-G) *Tg(flk1:egfp)* labels blood vessels in green; magenta labels cerulean-tagged *Mycobacterium marinum* (*Mm-cerulean*), which lies within a granuloma. Imaging commences at ∼400 µm below the scales; stack is ∼104 µm deep. Scale bar: 100 µm. Single Z frames were exported and gamma adjusted in FIJI/ImageJ for increased visibility, with all gamma adjustments applied uniformly across all images.

Work in larval zebrafish and in sectioned adults has demonstrated that infection with *M. marinum*, a close genetic relative of *M. tuberculosis*, recruits surrounding vasculature to the nascent granuloma (Oehlers et al., 2015). Vascular recruitment to *M. marinum* granulomas is crucial to bacterial growth; inhibition of granuloma vascularization with host-directed therapies, including VEGF inhibitors alone or together with established anti-tuberculosis drugs, limits bacterial proliferation (Oehlers et al., 2015). We used CLARITY to visualize this process in intact adult *Tg(flk1:egfp)* zebrafish infected with *M. marinum* expressing cerulean-fluorescent protein. We identified granulomas by the bacterial-laden central core formed in these structures (Fig. 3). As we had shown previously in larvae and in adult sections, we saw a strong association of blood vessels with tightly formed adult granulomas (Fig. 3). Consistent with our previous findings in larvae, the vascularization remained on the periphery and did not directly enter the granuloma (Matty et al., 2015; Oehlers et al., 2015). However, compared to sections of adult granulomas (Oehlers et al., 2015), we were able to visualize a much closer association of the vasculature, as well as define continuous vessels that encircled the mycobacterial granuloma (Fig. 3).

### PACT-based clearing of whole animals

A complementary technique to CLARITY, called PACT, has recently been described (Yang et al., 2014). This technique facilitates rapid passive clearing of tissues by fixing tissue prior to acrylamide embedding and removing bis-acrylamide from the hydrogel solution. Although initial tests of PACT by Yang et al. (2014) involved clearing of individual organs, PACT was applied to whole animals only via a perfusion-mediated process. We applied the solutions used in the PACT technique to the clearing of whole zebrafish without perfusion, reasoning that the relatively small size of adult zebrafish would render perfusion unnecessary. We found that PACT was able to clear animals comparably to our modified CLARITY technique, enabling whole-animal imaging. In *Tg(flk1:egfp)* zebrafish, we found that this technique behaved similarly to the low-acrylamide CLARITY solution we had used previously (Movie 3). We then adopted the PACT approach as a standard approach because of the simplified solution scheme used for this technique (see Materials and Methods section).

### Heterogeneity in TNF induction within the mycobacterial granuloma

**Fig. 4.**
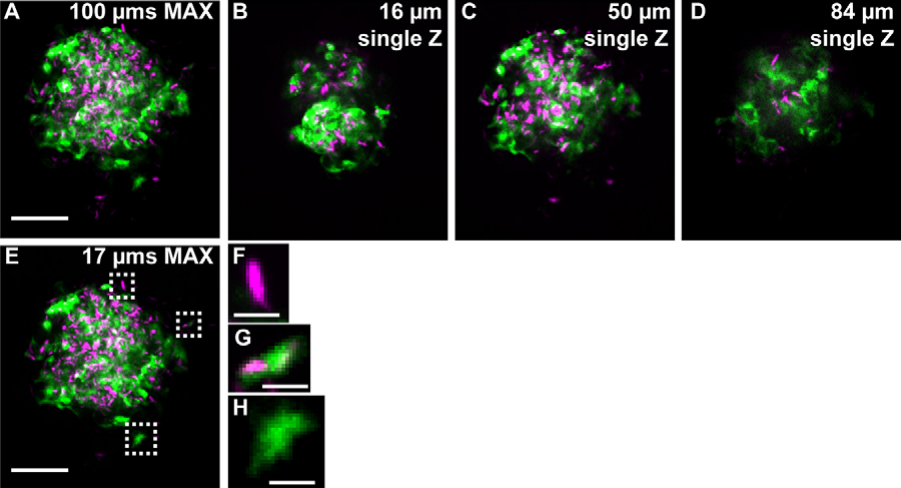
**Fluorescent mycobacteria and cytokine induction can be imaged deep within intact adult zebrafish.** (A) The TNF reporter (green) is expressed throughout a large granuloma (tdTomato-expressing *M.* *marinum*: magenta) in the *TgBAC(tnf:GFP)* line. Imaging begins 256 µm below the scales and the stack (A) is ∼100 µm deep; individual Z planes from the stack (B-D) reveal TNF reporter intensity throughout the granuloma. (E-H) TNF reporter expression in the granuloma is not dependent on infection status of individual cells: (F) an infected cell that does not express the TNF reporter; (G) an infected cell expressing the TNF reporter; (H) an uninfected cell expressing the TNF reporter. Scale bars: 50 µm (A-E); 5 µm (F-H). Single Z frames were exported and gamma adjusted in FIJI/ImageJ for increased visibility, with all gamma adjustments applied uniformly across all images.

Levels of the cytokine tumor necrosis factor (TNF) mediate the outcome of mycobacterial infection in zebrafish and mammalian models (Flynn et al., 1995; Keane et al., 2001; Gomez-Reino et al., 2003; Clay et al., 2008; Lin et al., 2010; Tobin et al., 2012; Roca and Ramakrishnan, 2013). In the zebrafish, diminished or elevated levels of TNF enhance bacterial growth through distinct mechanisms (Clay et al., 2008; Tobin et al., 2012; Roca and Ramakrishnan, 2013). In order to directly visualize the induction of this key cytokine and its spatial distribution within the granuloma, we used PACT in conjunction with the previously characterized TNF reporter line *TgBAC(tnf:gfp)*^pd1028^ (Marjoram et al., 2015). After infection with tdTomato-expressing *M. marinum*, we found that the TNF reporter was active in cells within and directly surrounding the bacteria-laden core of the granuloma (Fig. 4A-E). However, reporter expression was intermittent within the granuloma, indicating that maximal TNF induction is not a strictly cell-autonomous process but could depend on local and spatial cues. Indeed, the TNF reporter was active in both infected and uninfected cells (Fig. 4F-H). The variation in cytokine expression levels within granuloma macrophages is consistent with findings in tissue sections from macaque *M. tuberculosis* granulomas, demonstrating extensive heterogeneity in immune cell markers and inflammatory state (although TNF was not directly examined) (Mattila et al., 2013).

### PACT-based imaging of *M. tuberculosis* in mouse lung

**Fig. 5.**
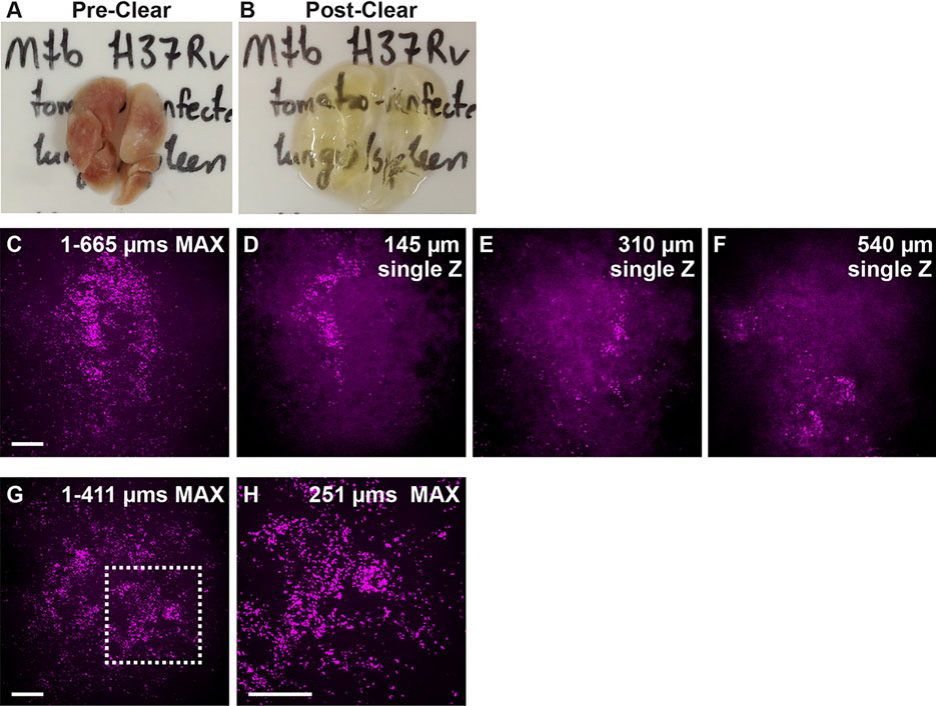
**Infection with fluorescent *M.tuberculosis* is visible throughout PACT-cleared mouse lungs.** (A,B) Mouse lungs pre- and post-clearing. (C-F) Stack through lung infected with approximately 350 CFU tdTomato-expressing *M. tuberculosis* at 28 dpi taken on Spinning Disc Confocal ranges from the top to bottom surface of a lung lobe (surface=1 µm) to 665 µm deep with 20× objective. (C) 665 µm maximum projection image. (D-F) Individual Z planes from stack. (G) 411 µm maximum projection image taken with 10× objective; (H) detail of boxed region from G taken with 20× objective; 251 µm maximum projection image. Scale bars: 100 µm. Single Z frames were exported and gamma adjusted in FIJI/ImageJ for increased visibility, with all gamma adjustments applied uniformly across all images.

We investigated whether these clearing techniques could be used to visualize infection in other model systems. The diverse array of immunological tools in the mouse has led to its use as an important model for *M. tuberculosis* infection. To determine whether we could visualize *M. tuberculosis* infection in intact lungs, we infected C57BL/6 mice with *M. tuberculosis* expressing the red fluorescent protein tdTomato. After fixation and removal from Biosafety Level 3 (BSL-3) conditions, lungs were subsequently cleared by the PACT technique and imaged. We observed numerous infecting mycobacteria spread throughout the lung (Fig. 5). As observed in tissue sections, granulomas in C57BL/6 mice are more diffuse than human granulomas (Flynn, 2006) but, even after fixation, the bacterial fluorescence is sufficient to enable high-resolution imaging of three-dimensional spatial distribution of infection within an intact lung. To confirm that autofluorescence from bacteria or lung tissue did not contribute to our signal, we cleared lungs from animals infected with non-fluorescent *M. tuberculosis* H37Rv (Fig. S1). Lungs infected with non-fluorescent *M. tuberculosis* were devoid of the characteristic fluorescent bacilli that are readily apparent in lungs infected with tdTomato-expressing *M. tuberculosis*. These findings indicate that the CLARITY and PACT techniques will be useful for imaging infection within tissues of mammalian model organisms.

## DISCUSSION

The recognition of infecting microbes by the host immune system results in the recruitment of diverse immune cell types. These host-immune interactions occur within varied, three-dimensional tissues and can result in the formation of complex structures. Yet, visualizing these host-microbe interactions in intact tissue remains difficult. Here, we demonstrate that the recently described CLARITY and PACT techniques enable high-resolution imaging of infection in whole zebrafish with *M. marinum* and in intact mouse lung tissue with *M. tuberculosis*. Using these techniques, we were able to image infection to a depth in excess of 1 mm within cleared whole zebrafish with little loss of resolution. This approach enables whole-animal analysis in adult zebrafish of vascularization and cytokine expression within granulomas. Both of these processes are critical to infection outcome, but their three-dimensional architecture has been relatively inaccessible in adults.

By using PACT in whole animals or modifications of CLARITY, we were able to facilitate rapid, passive clearing of tissue. The passive clearing technique enables deep visualization within cleared tissues without the damage that can occur with active clearing techniques (Tomer et al., 2014; Yang et al., 2014). Additionally, the passive clearing technique requires little equipment and minimal hands-on processing time, facilitating its use in labs with little setup. The relatively small size of adult zebrafish permits clearing to occur efficiently without perfusion, enabling imaging within intact animals without the need to dissect out individual organs. The technique is also compatible with *post hoc* dissection of organs of interest.

A number of cell-type- and cytokine-specific reporters have been described for zebrafish that enable the rapid visualization of cells and immune signals *in vivo* (Langenau et al., 2004; Renshaw et al., 2006; Hall et al., 2007; Ellett et al., 2011; Page et al., 2013; Palha et al., 2013; Marjoram et al., 2015). Gene-editing techniques in zebrafish, including TALENs and CRISPRs, have been demonstrated to allow insertion of reporters into endogenous genes using either homologous recombination or non-homologous end-joining (Chang et al., 2013; Auer et al., 2014; Irion et al., 2014; Shin et al., 2014; Hisano et al., 2015). These genome-editing techniques will allow the generation of new transgenic lines for studying immune signaling processes *in vivo*. Cleared tissue is compatible with immunostaining, eventually enabling the use of validated antibodies within infected animals and organs (Chung et al., 2013; Tomer et al., 2014; Yang et al., 2014). The CLARITY and PACT techniques have also been demonstrated to retain RNA molecules, enabling *in situ* detection of RNA (Chung et al., 2013; Yang et al., 2014). Together with CLARITY and PACT, these techniques will enable whole-animal visualization of the cell types and signaling molecules participating in host immune responses to microbial infection.

## MATERIALS AND METHODS

### Zebrafish handling

All zebrafish (*Danio rerio*) husbandry and experimental procedures were performed in accordance and compliance with policies approved by the Duke University Institutional Animal Care and Use Committee (protocol A145-14-06). Strains include *Tg(kdrl:egfp)^s843^*, referred to as *Tg(flk1:egfp)* (Jin et al., 2005), *TgBAC(tnfa:GFP)^pd1028^* (Marjoram et al., 2015), *Tg(Xla.Tubb:DsRed)^zf148^* (Peri and Nüsslein-Volhard, 2008) and *Tg(LysC:DsRed)^nz50^* (Hall et al., 2007).

### Adult zebrafish infection

Adult zebrafish were anesthetized with tricaine (MS-222; Sigma-Aldrich; final concentration 160 μg ml^−1^) and infected with approximately 500 CFU of fluorescent *M. marinum* via intraperitoneal injection. *M. marinum* strains tagged with either cerulean or tdTomato have been previously described (Takaki et al., 2013; Oehlers et al., 2015). Zebrafish were maintained in beakers in a dedicated incubator at 28°C with a 14:10 h light:dark cycle for 2 weeks until euthanized.

### Mouse infection and organ processing

Four C57BL/6 female mice were aerosol-exposed to approximately 350 CFU *M. tuberculosis* H37Rv:tdTomato or H37Rv (non-fluorescent) per animal as previously described (Saini et al., 2011; Smith et al., 2014b). All animal studies were approved by the Institutional Animal Care and Use Committee of Duke University (protocol A065-13-03). Following euthanasia at 28 days post-infection, the lungs, liver and spleen were excised, fixed in 10% neutral buffered formalin for 24 h, transferred to 70% ethanol and rehydrated prior to hydrogel embedding.

### CLARITY and PACT clearing

CLARITY was performed by modifying the acrylamide concentration used in the protocol described in Chung et al. (2013) to 1%. Adult zebrafish were incubated for 3 days in a solution of 4% PFA, 1% acrylamide, 0.05% bis-acrylamide and 0.25% photoinitiator 2,20-Azobis[2-(2-imidazolin-2-yl) propane]dihydrochloride (VA-044, Wako Chemicals USA) in 1× phosphate buffered saline (PBS) at 4°C. Hydrogel solutions were overlaid with mineral oil and the hydrogel was polymerized by incubating for 3 h at 37°C. Tissue was removed from excess hydrogel and tissue was incubated in 4% SDS in 200 mM boric acid, pH 8.5 at 37°C with shaking, and the SDS solution was changed every other day. Clearing of the tissue was achieved in 7-10 days. After clearing, the tissue was washed twice for 1 day in PBS, 0.1% Triton X-100 at 37°C. Refractive-index matching was achieved by incubating the cleared tissue in RIMS solution (Yang et al., 2014).

PACT clearing was performed based on the approach of Yang et al. (2014) except that the solutions were applied by soaking rather than perfusion-driven processes. This modification resulted in alterations in the timing of the incubation steps. Briefly, zebrafish were fixed in 4% paraformaldehyde (PFA) for 2 days at 4°C. Mouse lungs were fixed in 10% neutral buffered formalin for 24 h, transferred to 70% ethanol and rehydrated prior to hydrogel embedding. Fixed whole adult fish and mouse lungs were incubated at 4°C for 3 days in the freshly made hydrogel monomer solution of A4P0 (4% acrylamide in PBS) supplemented with 0.25% VA-044. A4P0-infused samples were incubated for 3 h at 37°C to initiate tissue-hydrogel hybridization. Fish were next removed to clean 15 ml conical tubes and incubated in 8% SDS in 200 mM boric acid, pH 8.5, for 5 days at 37°C with shaking, and SDS solution was changed every other day. Samples were then washed for 1 day each in two changes of PBS, 0.1% Triton X-100 at 37°C, then incubated in RIMS imaging media (Yang et al., 2014) for 1 day at room temperature on a rotator. Samples were stored in RIMS at room temperature.

### Fluorescence microscopy

Cleared tissue samples were mounted in RIMS at room temperature in MatTek dishes and held in place using modeling clay, then imaged as indicated on one of three microscopes, as described in the relevant figure legend. Images were acquired with an Olympus FV1000 multiphoton system with a 25×/0.9 NA ScaleView immersion lens [XLPLN25XSVMP 8 mm working distance (WD) and 0 to 0.23 μm coverslip correction] immersed in SCALE reagent, with an Andor XD spinning disc confocal microscope with a 10×/0.3 UPlanFl N dry, WD: 10 mm, FN26.5, UIS2 objective and a 20×/0.5 UplanFl N dry, WD: 2.1 mm, ∞/0.17/FN26.5, UIS2 objective, or acquired on a Leica SP8 confocal with a 25×/0.95 HCXIRAPO water dipping lens. Epifluorescence imaging was performed on a Zeiss Axio Observer.Z1 fitted with a Zeiss Fluor 5×/0.25 lens, a 0.5× C-Mount and a Zeiss AxioCam MRm. After imaging, samples were returned to RIMS at room temperature for storage.

### Image processing

Image stacks were compressed into maximum intensity projections (MIPs) in their respective acquisition software package, or individual frames were selected. MIPs and single Z frames were exported and gamma adjusted in FIJI/ImageJ for increased visibility, color assigned by acquisition wavelength, and analyzed, with all adjustments applied uniformly to each image. Brightness, contrast and color levels were adjusted in Adobe Photoshop.
